# Advice upon Medical Subjects

**Published:** 1869-04

**Authors:** 


### Advice Upon Medical Subjects.

BY THE EDITOR.

BLACK EYE.—Every one is anxious to dispose of a "black eye" as speedily as possible, doubtless for the reason that it is the insignia of the ruffian and loafer. All sorts of plans are resorted to for the purpose of accomplishing this desirable end. Rotten apples, raw meat, mud, pounded onion, sour-krout, &c., &c., are popular means of relief; but we hope to spare our readers from all such disagreeable applications, by assuring them that there is no virtue whatever, in this mess of stuff.

Fomentation with cloths saturated with hot water, is the means for hastening the absorption of the effused blood under the skin. If pain accompanies the bruise, a half ounce of laudanum, added to a pint of hot water and applied to the bruise by means of the cloths, will give relief. The water should be as hot as can be borne, and must be applied constantly for six or eight hours to have any beneficial effect. You will then have the pleasure of seeing the blue-black change to a beautiful copper color, and finally to a dirty yellow, several days being necessary for dame nature to restore the parts to their original hue.

BROKEN BONES.—Much pain and suffering might be spared the patient with a broken arm or leg, it the bystanders would observe the following rules: Select a small tree, about the size of the broken limb. Remove the bark in two sections, and of the same length as the broken bone. Then, supposing the thigh to be broken, place some cotton wool in one of the pieces of bark, and place it under the limb. Let some strong man take hold of the heel and top portion of the foot, and pull with great force, while another person holds back the body of the patient by means of a towel placed between patient's legs and drawing toward his head. Let a third person feel of the bone at the seat of fracture, and direct those who are extending the limb, how hard to pull in order to have the parts assume their proper position. This may be told by the bone feeling smooth, where it was lapped before. Next adjust the other half, of the bark, well padded, to the other side of the limb, and around all wind a bandage as snugly as possible. Lay your patient upon a lounge, place an old shoe upon his foot, to the middle of the sole of which attach a stout cord, by running it between the sole of the shoe and the foot. Tie two smoothing irons or other weights to the end of the cord, and let them hang over the end of the lounge in such a manner as to keep a constant extension upon the limb in a direct line with the body. If you have been successful in adjusting the fracture, when the physician arrives, it will only be necessary for him to satisfy himself of the fact, and no tugging and pulling will be required when the limb has become sore, swollen and inflamed.

In cases of railroad accidents, or those occurring in the country, far remote from a surgeon, these suggestions will enable almost any one to improvise a capital splint and successfully adjust a fracture.

---

CROUP.—Parents should watch their little ones carefully at this season of the year for any symptoms of croup; as the sudden changes that are liable to occur in the spring time, from warm to cold, will expose our little folks to the danger of contracting "colds," which are so readily converted into that dreaded disease—croup.

Have your children comfortably clad with woolen underclothing, and rubber overshoes. Do not allow them to wear scarfs or mittens at this time of year, for they are soon thrown off in the heat of play, and so easily contract "colds."

Always have some unslacked lime among your "family medicines;" and should the child have a cough, and a sort of wheezing in breathing, rub its breast well with sweet oil, bathe its feet in hot water, and put it to bed. Upon the first appearance of that hollow, croupy cough, let it inhale the vapor from a piece of the lime placed in a dish of hot water. This will at once give relief and prevent the further progress of the disease.

---

FELONS—Not the sort to be found in our County Jail, but the kind that are confined to your fingers. Felons are serious affairs oftentimes. The best thing to be done is to have the finger opened at once. Do not wait for it to "come to a head." Therein lies the danger. The disease exists in the covering membrane of the bone, (*periosteum*,) and, unless you give the pus free means of escape, it will destroy the surrounding tissue, and involve the bone in the trouble. Unsightly deformities of the fingers and hands are often caused by the neglect to open a felon in season.

As soon as satisfied you have a "felon," go to your physician and have him lay the parts open to the bone; then apply a hot poultice of bread-and-milk. This course, if adopted *at once*, will spare you much pain and trouble.

---

"RING WORM."—(*Herpes Circinatus*)—This is a disease of the skin usually occurring about the face and shoulders. It makes its appearance in the form of an eruption, composed of numerous little round vesicles or bladders arranged in a circular form, and containing a milky sort of fluid. The skin is of a vivid red color and highly irritable. The disease usually runs its course in eight or ten days, terminating in a scabby condition of the parts, which soon drop off, leaving the skin smooth as before. Sometimes the disease assumes a chronic character, when the following ointment may be applied, which will speedily effect a cure:

℞.

Cupri Sulphas Pulv. gr. x.

Simple Cerate ʒ. j.

Fiat ung:

Apply to the diseased skin once every day.

---

SCROFULOUS SWELLINGS.—When the glands about the neck become painful and swollen, or the ears are occasionally "bealing;" have the following prescription prepared at your druggist's:

℞.
Oli Morrhuæ. O. j.
Tinct. Ferri Chloridi, ℥. ij.
Misce.

And take, for an adult, one teaspoonful three times per day. The mixture must be thoroughly shaken before every dose is poured from he bottle.

This prescription will be found valuable in all manner of *scrofulous* diseases.

---

SORE THROAT.—Touch the inflamed throat and tonsils with a solution of ten grains of *Carbolic Acid*, in one ounce of water. Use a camel's hair brush, with long handle, for the solution, and a spoon handle to depress the tongue with. Bind a piece of flannel, saturated with kerosene oil, about the neck at night. Follow up this treatment for about one week, and you can cure the most obstinate sort of sore throat.

---

MEASLES.—Very little treatment is necessary for children with measles. Indeed the less you do for them in ordinary cases, the better. If the fever runs high, and the child is restless, and complains of itching of the skin, a hot vinegar sponging of the entire body may be resorted to; being very careful however, that the room be warm, and no drafts of air reach the bedside. After the bath wrap the child in flannel and administer some warm drink. It will then soon fall into a refreshing sleep.

Slippery elm water may be given to drink, in order to allay the disposition to cough.

---

COUGH MIXTURE IN CHRONIC BRONCHITIS.

℞

Linseed Oil.
Simple Syrup āā ℥. j.
Ess. Peppermint gtt. jv.
Mix.

A desert spoonful three times per day.

This will be found an excellent mixture for the many persons we find at this season of the year, with the above trouble.

---

"SORE EYES" OF INFANTS.—Babies, when two or three days old, are often troubled with an inflammation of the eyes. Yellow matter is found between the lids, which are kept tightly closed, and often greatly swollen.

Wash the matter from the eyes, with warm water and castile soap, then procure one drop of the *Solution of Carbolic Acid*, in one ounce of Rose Water. With this, pencil baby's eyes three times per day, and you will have the satisfaction of seeing the disease subside.

---

**Whooping Cough.**

The late Dr. Valentine Mott's prescription was as follows:

℞

Hydrocynic acid, gtt. vj.
Ext. Belladonna, gr. ij.
Paregoric, ʒ iij.
Syrup Balsam of Tolu, ℥ j.
Aqua, ℥ iij.

M. S.—One teaspoonful four times daily.

---

### Strychnine in Beer.

It is said, in the Medical Journals of London, that strychnia is being employed instead of hops to impart the bitter to pale ale, made in that city. Wonder how long ere the poison will be used in this country? Beer drinkers should be careful to use that only from reliable manufacturers.

